# A Review on the Role of Oral Bacteria in Stroke

**DOI:** 10.3390/ijms262411913

**Published:** 2025-12-10

**Authors:** Florencia Gayo, Jorge Moldes, Susana Bravo, Irene Vieitez, Lucía Martínez-Lamas, Manuel Rodríguez-Yáñez, Ramón Iglesias-Rey, Pedro Diz, Tomás Sobrino, Juan Blanco, Yago Leira

**Affiliations:** 1 Unit of Periodontology, Faculty of Dentistry and Medicine, University of Santiago de Compostela, 15705 Santiago de Compostela, Spain; 2 Medical-Surgical Dentistry Research Group (OMEQUI), Health Research Institute of Santiago de Compostela (IDIS), 15706 Santiago de Compostela, Spain; 3 Family and Community Medicine Department, Bueu Health Centre, Pontevedra University Clinical Hospital, 36071 Pontevedra, Spain; 4 Proteomic Unit, Health Research Institute of Santiago de Compostela (IDIS), University Clinical Hospital, 15706 Santiago de Compostela, Spain; 5 Genomic Unit, Galicia Sur Health Research Institute (IIS Galicia Sur), SERGAS-UVIGO, 36312 Vigo, Spain; 6 Department of Microbiology and Parasitology, Complexo Hospitalario Universitario de Santiago, 15706 Santiago de Compostela, Spain; 7 Microbiology Group, Health Research Institute of Santiago de Compostela (IDIS), 15706 Santiago de Compostela, Spain; 8 Stroke Unit, Neurology Department, University Clinical Hospital, 15706 Santiago de Compostela, Spain; 9 Neuroimaging and Biotechnology Laboratory Group, Clinical Neurosciences Research Laboratories, Health Research Institute of Santiago de Compostela (IDIS), University Clinical Hospital, 15706 Santiago de Compostela, Spain; 10 Special Care Unit, Faculty of Dentistry and Medicine, University of Santiago de Compostela, 15705 Santiago de Compostela, Spain; 11 NeuroAging Group (NEURAL), Clinical Neurosciences Research Laboratory (LINC), Health Research Institute of Santiago de Compostela (IDIS), 15706 Santiago de Compostela, Spain; 12 Center for Networked Biomedical Research on Neurodegenerative Diseases (CIBERNED), Carlos III Institute of Health, 28029 Madrid, Spain

**Keywords:** bacteria, cerebral ischemia, periodontitis, stroke

## Abstract

Emerging evidence suggests periodontitis may contribute to stroke risk via vascular inflammation and endothelial dysfunction, promoting atherothrombosis and atrial fibrillation. This review aims to synthesize the evidence on the presence of oral bacteria and their products in biological samples from stroke patients and assess their potential impact on stroke pathophysiology, clinical outcomes, and prognosis. We conducted a narrative review of epidemiological, serological, and molecular studies examining the presence of oral bacterial DNA, endotoxins and antibodies against oral pathogens in biological samples (blood, saliva and thrombi) from stroke patients. Seropositivity for periodontal pathogens in blood was associated with incident stroke, as well as with poorer prognosis. Oral bacterial DNA, mainly from *Streptococcus* spp. and *Prevotella* spp., was consistently detected in thrombi, whereas no DNA from classic periodontal pathogens was found. The presence of *P. gingivalis* antibodies in thrombi was associated with lower complete reperfusion rates, while *Acinetobacter* spp. and *Enterobacteriaceae* correlated with higher early adverse events and poorer prognosis. DNA detection was limited by low-biomass samples and methodological constraints. These findings support a potential link between periodontitis and ischemic stroke. However, further studies using improved molecular methods are needed to clarify underlying mechanisms and to assess the presence of periodontal pathogen DNA in thrombi.

## 1. Background

Stroke is characterized by the abrupt onset of a focal neurological deficit resulting from a vascular disturbance, either due to an interruption of cerebral blood flow (ischemic stroke) or to the rupture of a cerebral vessel (hemorrhagic stroke) [1]. The former accounts for 65.3% of all incident cases, whereas the latter represents 34.6% [2]. Overall, stroke is associated with significant global morbidity and mortality, which has been increasing over the past 30 years [2]. Currently, it is the second leading cause of mortality worldwide and the third of combined death and disability, resulting in the loss of approximately 160 million disability-adjusted life years (DALYs) [2]. Moreover, these figures are projected to worsen over the next 25 years, with annual deaths expected to reach 9.7 million, primarily driven by its growing incidence in low- and middle-income countries [2]. Stroke is a highly preventable condition, with 84% of cases considered attributable to 23 well-established modifiable risk factors, including major contributors such as smoking, hypertension, physical inactivity, and obesity [2]. In this context, additional potential risk factors for stroke have been explored, among which periodontitis has gained increasing attention.

Periodontitis is a chronic, multifactorial inflammatory disease involving the progressive breakdown of the tooth-supporting tissues, including periodontal ligament and alveolar bone [3]. It is caused by the proliferation of some oral bacteria inducing dysbiosis with biofilm formation, which damages the periodontal supporting tissues and triggers a local inflammatory response that exacerbates and perpetuates tissue destruction [3]. Diagnosis is established by demonstrating tissue damage, typically through the measurement of clinical attachment loss (CAL) [4]. However, not all individuals with bacterial plaque accumulation develop the disease, as its onset depends on the interaction between microbial biofilm, environmental influences, and individual susceptibility [3].

Over the past three decades, periodontitis has shown a significant global increase in incidence, prevalence, and DALYs [5]. This rise has been particularly pronounced in regions such as Sub-Saharan Africa and Southeast Asia, highlighting an inverse relationship between disease burden and socioeconomic development. While the highest burden is observed among individuals aged 55 to 59 years, there is a growing trend towards earlier onset, with a sustained increase in cases among individuals under 38 years of age [5]. Regarding sex distribution, data do not reveal significant differences between men and women. These findings underscore the need to recognize periodontitis as a global and chronic public health issue, requiring comprehensive prevention and management strategies, particularly in low- and middle-income countries and under-resourced areas [5].

Beyond its increasing epidemiological burden, periodontitis has gained attention for its systemic implications. Robust scientific evidence has revealed strong associations between periodontitis and a broad spectrum of systemic conditions [6], including various types of cancer, cardiovascular diseases, metabolic disorders, immune-mediated diseases, respiratory disorders, adverse pregnancy outcomes, neurodegenerative disorders and cerebrovascular diseases [6].

The association between periodontitis and stroke has been well documented through multiple systematic reviews [7,8]. More recently, an umbrella review has synthesized most of the available evidence, providing a comprehensive overview of the consistency and strength of this relationship [9]. The different types of strokes have been analysed separately, with the association persisting in both ischemic [10,11] and haemorrhagic stroke [12]. However, the link between periodontitis and ischemic stroke—particularly the atherothrombotic and cardioembolic subtypes—has been more extensively studied, given the stronger biological plausibility through atherosclerosis and atrial fibrillation (AF) [13].

Atherosclerosis is a chronic inflammatory disorder of the vasculature that has been closely associated with periodontitis [14]. These two conditions have been linked through three biological mechanisms capable of inducing endothelial dysfunction and vascular inflammation, thereby promoting the initiation, progression, and rupture of atherosclerotic plaques [15,16]. First, direct bacterial invasion of the vascular endothelium has been proposed as a key mechanism mediating the relationship between periodontitis and atherosclerosis. Several studies have detected the presence of periodontal pathogens and their products within atherosclerotic plaques [17], and some have even demonstrated their viability [18,19]. The most frequently isolated periodontal bacteria include *Porphyromonas gingivalis*, *Aggregatibacter actinomycetemcomitans*, *Fusobacterium nucleatum*, *Prevotella intermedia*, *Tannerella forsythia*, *Treponema denticola*, and *Campylobacter rectus* [20]. Each of these species expresses specific virulence factors—such as lipopolysaccharides, fimbriae, and proteases—that permit evasion of the host’s immune defenses, facilitate adhesion to and invasion of endothelial cells, dysregulate lipid metabolism and trigger inflammatory cascades [20]. Additionally, higher rates of bacteraemia involving these pathogens have been observed in patients with periodontitis [21,22,23] and indirect transport via dendritic cells has also been described [24,25]. Secondly, the development of low-grade chronic systemic inflammation has emerged as another potential pathway linking the two conditions. This process may originate from the translocation of oral bacteria, their by-products, inflammatory cells, and proinflammatory cytokines into the bloodstream through ulcerated or damaged periodontal tissues [26,27,28]. Once in systemic circulation, these mediators may promote the transition from a localized to a systemic inflammatory response. Supporting this hypothesis, robust scientific evidence has demonstrated a positive correlation between periodontitis and elevated levels of inflammatory biomarkers, such as C-reactive protein (CRP) [29,30], tumor necrosis factor-alpha (TNF-α) [31,32], and interleukins 1β and 6 (IL-1β, IL-6) [33], which tend to decrease following periodontal treatment [34,35,36]. This systemic inflammatory state has been proposed as an etiological factor in atherosclerosis, due to the critical role of vascular inflammation in the initiation, progression, and rupture of atheromatous plaques [28,37,38,39]. In this context, several studies have identified a correlation between serum levels of CRP [40,41,42] and IL-6 [43,44,45,46] and atheroma progression assessed by imaging techniques and hemodynamic functional tests. Thirdly and finally, some studies have reported antibodies against antigens from *Porphyromonas gingivalis*, *Aggregatibacter actinomycetemcomitans*, *Fusobacterium nucleatum*, and *Tannerella forsythia* that cross-react with vascular endothelial antigens such as heat shock proteins (HSPs), cardiolipin, modified lipids, and apolipoprotein A1 [16]. This molecular mimicry induces endothelial activation and dysfunction, promotes the production of inflammatory cytokines, and triggers the migration of monocytes into the subendothelial space, thereby favouring the development of atheroma [16]. Furthermore, inflammation resulting from any of the three mechanisms described promotes the development of a prothrombotic state [47,48], ultimately potentiating thrombosis following the rupture of the atherosclerotic plaque.

AF has also been found to be significantly associated with periodontitis [49]. Moreover, Park et al. observed that patients with chronic periodontitis exhibited a higher incidence of AF compared to those with newly diagnosed periodontitis, and that the resolution of periodontitis reduced the risk of AF, although not to the level observed in individuals without a history of periodontitis [50]. Additionally, periodontal treatment and oral health interventions have been shown to lower both the incidence and recurrence of AF [51,52]. This association between AF and periodontitis may be explained by the same mechanisms previously described in the link between periodontitis and atherogenesis. Direct bacterial invasion has recently been investigated by Miyauchi et al., who identified the presence of *Porphyromonas gingivalis* within the atrial tissue of mice with experimentally induced periodontitis [53]. Their work established both qualitative and quantitative associations between bacterial presence, the degree of atrial fibrosis and the onset of AF [53]. Previously, the same group had reported an association between periodontitis and atrial fibrosis in humans [54]. In parallel, the role of inflammation in the pathophysiology of atrial fibrillation has been well established through its contribution to atrial electrical and structural remodeling [55]. In this regard, the influence of systemic inflammation on AF has been demonstrated through several biomarkers [56]—particularly interleukin-6 (IL-6)—as well as inflammatory indices [57]. Finally, a recent systematic review has demonstrated an association between the levels of various autoantibodies (including anti-β, anti-M2, anti-HSP, and others) and the incidence of AF [58], suggesting a potential role of molecular mimicry and autoimmunity in its development.

To date, no review has specifically focused on oral bacteria and their potential effects on cerebral ischemia. In this context, the present review aims to synthesize the available evidence on the presence of bacteria and their products in a range of biological samples from stroke patients, including blood and thrombotic material, thereby providing an updated and comprehensive overview of their potential contribution to ischemic cerebrovascular disease.

## 2. Oral Bacteria and Stroke

### 2.1. Antibodies Against Periodontal Pathogens in Stroke

Several studies investigated the association of stroke with serum levels of specific antibodies against different periodontopathogens [59,60,61,62,63,64,65,66,67,68,69,70,71,72].

A nested case–control study by Pussinen et al. showed that subjects seropositive for immunoglobulin (Ig) A against *A. actinomycetemcomitans* and *P. gingivalis* were more likely to develop a first-ever stroke (adjusted OR = 1.7; 95% CI = 1.0–2.9) or a recurrent stroke (aOR = 2.6; 95% CI = 1.0–7.0), respectively [59]. The same Finnish research group further reported a positive association between serum IgA (in men) and IgG (in women) specific to *P. gingivalis* and incident stroke in a 15-year follow-up study (aOR = 1.6; 95% CI = 1.1–2.5 and aOR = 2.3; 95% CI = 1.4–3.8, respectively) [60]. Other studies, mainly from Asia, found similar results in relation to the presence of serum IgG levels against periodontal bacteria and the risk of having cerebral infarction [62,65] or cerebral hemorrhage [69]. Recently, Hallikainen et al. found that high serum levels of IgA against *P. gingivalis* and *A. actinomycetemcomitans* were associated with increased risk of unruptured intracranial aneurysms (aOR = 1.4; 95% CI = 1.1–1.8 and aOR = 2.3; 95% CI = 1.7–3.1, respectively) and aneurysmal subarachnoid hemorrhage (aOR = 1.5; 95% CI = 1.1–1.9 and aOR = 2.1; 95% CI = 1.5–2.9, respectively) [73]. In contrast, high serum levels of IgG against *P. gingivalis* and *A. actinomycetemcomitans* were associated with lower risk of unruptured intracranial aneurysms (aOR = 0.6; 95% CI = 0.4–0.8 and aOR = 0.6; 95% CI = 0.4–0.7, respectively) and aneurysmal subarachnoid haemorrhage (aOR = 0.5; 95% CI = 0.4–0.7 and aOR = 0.6; 95% CI = 0.5–0.9, respectively) [73].

Stroke patients who are seropositive IgG subjects against several periodontopathogens are more prone to having poor functional outcome for: stroke (both types of stroke: aOR = 3.1; 95% CI = 1.5–6.3; ischemic stroke: aOR = 1.2; 95% CI = 1.0–1.4; and hemorrhagic stroke: aOR = 7.9; 95% CI = 1.1–57.1) [67,69,71], atrial fibrillation (ischemic stroke: aOR = 4.4; 95% CI = 1.7–12.1) [63], bulb/internal artery carotid atherosclerosis (ischemic stroke: aOR = 16.6; 95% CI = 4.0–78.9) [63] and cerebral microbleeds (both types of stroke: aOR = 2.0; 95% CI = 1.2–3.5) [70].

The role of antibodies against oral microorganisms in the infectious burden of stroke has also been explored. It was found that an infectious burden defined by cumulative IgA seropositivity to *A. actinomycetemcomitans*, *P. gingivalis*, *C. pneumoniae*, *Mycoplasma pneumoniae*, and *Helicobacter pylori* was associated with an increased risk for first-ever large-vessel ischemic stroke, but the finding was not independent of unfavorable childhood socio-economic conditions [66]. In a study recently published by the same research group, it was not possible to demonstrate a potential interaction between infectious burden, traditional cardiovascular risk factors and a more pro-inflammatory genetic profile that would increase the likelihood of having an ischemic stroke [72].

### 2.2. Endotoxemia and Stroke

Endotoxemia has also been studied in stroke. A nested case–control study using data from two Swedish epidemiological surveys (MONICA and VIP studies) reported a negative association between circulating *A. actinomycetemcomitans* leucotoxin-neutralizing antibodies with first-ever stroke in women (aOR = 0.3; 95% CI = 0.1–0.6) but not in men (aOR = 0.9; 95% CI = 0.5–1.5) [61]. Palm and co-workers measured lipopolysaccharide (LPS) activity in serum and saliva of ischemic stroke patients and healthy controls [64]. Concentrations of LPS in saliva but not in serum were higher in cases than in controls [64]. Similarly, a secondary analysis of the GenesiS study did not find differences in terms of serum levels of LPS-neutralizing capacity or in LPS activity between ischemic patients and controls [68]. However, both studies found increased salivary antibody levels against *A. actinomycetemcomitans* in ischemic stroke patients compared to controls [64,68].

### 2.3. Oral Bacterial DNA and Stroke

Oral bacteria have been analysed in thrombi from patients with ischemic stroke (Table 1).

Yadav et al. analysed thrombi from 14 patients with ischemic stroke. Bacterial DNA was detected in 100% of the thrombi thanks to 16S rRNA sequencing and metagenomic analysis, identifying over 30 bacterial species. Among the oral bacteria detected were *Streptococcus pneumoniae*, *Steptococcus pyogenes* and several *Prevotella*, *Lactobacillus*, *Veillonella* and *Sneathia*. Notably, patients with significantly higher levels of *Acinetobacter* spp. and *Enterobacteriaceae* exhibited an increased rate of early adverse events following reperfusion and poorer clinical prognosis [75].

Although bacterial DNA is found in thrombotic material from strokes, the proportion of specific oral bacterial traces is not that abundant. Liao’s research group, using 16S rRNA gene amplicon next-generation sequencing, found that oral bacterial DNA in 104 ischemic stroke thrombi accounted for only 2.3% of bacterial communities [76]. Consistent with Yadav et al., the presence of *Acinetobacter* spp. and *Enterobacteriaceae* in thrombi was associated with an increased risk of perioperative adverse events. Furthermore, higher abundance of *Acinetobacter* spp. was independently linked to an elevated 90-day mortality risk (HR: 2.02; 95% CI: 1.20–3.41; *p* = 0.008) [76].

There is a case–control study in which DNA from two major periodontal pathogens (*P. gingivalis* and *A. actinomycetemcomitans*) was quantified by conventional and real-time polymerase chain reaction (PCR) [80]. Subgingival plaque samples were collected from 20 cases (13 patients diagnosed with ischemic stroke and 7 with haemorrhagic stroke) and 60 healthy controls. Classical clinical periodontal parameters were also recorded. Results showed that DNA from *A. actinomycetemcomitans* was absent across all groups, either by conventional or real-time PCR. However, DNA from *P. gingivalis* was detected more often in stroke patients than in controls (using both methods). While a positive correlation was found between increased probing depth and the quantity of *P. gingivalis* DNA in ischemic stroke patients (r = 0.6, *p* = 0.03), no statistically significant correlation was observed in hemorrhagic stroke subjects (r = 0.2, *p* = 0.09) [80].

The presence of DNA from *P. gingivalis* and *A. actinomycetemcomitans* was also analysed in thrombi from 75 patients suffering from acute ischemic stroke who underwent mechanical thrombectomy [74]. Real-time quantitative PCR revealed no evidence of bacterial DNA from these two periodontopathogens in thrombus aspirates. In contrast, *Streptococcus* spp. DNA—mainly from the *S. mitis* group—showed a higher median value (5.10-fold) compared to control blood specimens from the same patients. Total bacterial DNA was also elevated (7.93-fold) relative to peripheral blood samples. A history of cerebrovascular disease was significantly more common among bacterial DNA–positive patients (*p* = 0.046) compared to those negative for bacterial DNA. However, there were no significant demographic differences between patients positive or negative specifically for *S. mitis* group bacterial DNA [74].

In a histological follow-up study [78], the same group performed an immunohistochemical analysis of the thrombus samples obtained in the “Brain, Microbes and Genetics” (BMG) project, from which the previous study was derived [74]. It was found that 84.8% of the samples were histologically positive for *Streptococcus viridans* group [78]. In the same study, carotid endarterectomy samples from two previous research projects were also analyzed. One cohort consisted of 20 patients with symptomatic carotid stenosis from the Tampere Vascular Study (TVS) and the other included 48 post-mortem carotid artery samples from individuals who had died suddenly in the Tampere Sudden Death Study (TSDS). The symptomatic cohort showed 80% of samples positive for *Streptococcus*, whereas the autopsy samples revealed only 31.3% positivity [78].

Wang et al. [77] further supported the presence of oral taxa in thrombi, studying through PCR the bacterial DNA composition of thrombi from 81 patients who had suffered acute ischemic stroke. Bacterial diversity in thrombi was significantly higher than in venous blood samples (*p* < 0.05), suggesting a greater bacterial presence within the clots. Oral-associated bacteria, including *Streptococcaceae* (mainly *Streptococcus* spp.), *Corynebacteriaceae*, and *Prevotella* spp., were significantly enriched in thrombi, with *Streptococcus* spp. present at 1.53% vs. 0.29% in arterial blood (*p* = 0.001) and *Prevotella* spp. at 1.57% vs. 0.38% (*p* = 0.010). Transmission electron microscopy revealed partial bacteria-like structures in 27.1% (22/81) and whole bacteria-like structures in 8.6% (7/81) of thrombi samples. Immunohistochemical staining for CD14, a monocyte/macrophage marker linked to bacterial presence, was positive in 63.0% of thrombi. Additionally, 38.5% of patients who tested positive for prokaryote-specific DNA reported alcohol consumption, compared to 16.4% of those negative (*p* = 0.03). After adjusting for age and sex, alcohol intake was significantly associated with a higher bacterial burden in thrombi (OR = 3.19; *p* = 0.033) [77].

Using real-time quantitative PCR, Pyysalo et al. analyzed samples from ruptured (*n* = 42) and unruptured (*n* = 28) intracranial aneurysms [81]. *F. nucleatum* was the most common periodontal bacterium found in both samples. DNA from *P. gingivalis*, *A. actinomycetemcomitans*, *T. denticola*, and *P. intermedia* was much less prevalent [81]. Previously, the same group was the first to detect DNA from periodontopathogens in intracranial aneurysm walls from 36 subjects diagnosed with subarachnoid hemorrhage (specimens were taken from 7 autopsy cases and from 29 patients who underwent surgical treatment) [82]. Recently, the same research group confirmed the presence of virulence factors from *P. gingivalis* (lipopolysaccharide and gingipain) in tissue samples from two patients with unruptured intracranial aneurysms [73].

The presence of *P. gingivalis* was also studied in thrombi from stroke patients retrieved through thrombectomy [79], finding that specific immunostaining was positive in 33.7% of thrombi (95% CI: 26.7–40.8%). Moreover, the analysis helped to characterize the 175 consecutive thrombi retrieved: patients with *P. gingivalis*-positive thrombi were associated with greater tooth loss compared to the *P. gingivalis*-negative group (median: 5 vs. 3). No significant differences were observed between groups regarding pre-stroke disability, stroke severity, or stroke aetiology. However, *P. gingivalis*-positive thrombi were associated with a lower rate of complete reperfusion after treatment (39.0% vs. 57.8%) and higher levels of neutrophil elastase (median: 180 vs. 129). There was also a trend towards worse functional outcomes at 90 days in the *P. gingivalis*-positive group compared to the *P. gingivalis*-negative group, although this difference did not reach statistical significance [79].

Taken together, these studies indicate that while oral bacteria are variably detected depending on method sensitivity and target species, multiple independent groups have confirmed their presence in thrombi from ischemic stroke patients. The data suggest potential clinical relevance, as certain bacteria could correlate with procedural difficulty and possibly worse outcomes. However, methodological heterogeneity among studies limits the comparability of findings and the robustness of conclusions. Metagenomic techniques based on 16S rRNA gene sequencing present important limitations when applied to low-biomass samples [83]. Moreover, the taxonomic resolution of 16S rRNA is often insufficient to discriminate between closely related species or pathogenic and non-pathogenic strains, thereby limiting its ability to accurately identify the microorganisms involved [84].

Detection of oral bacteria in thrombi presents significant analytical challenges. Low DNA concentrations often fall below the detection threshold of standard qPCR, a limitation compounded by the presence of endogenous inhibitors such as heparin or hemoglobin in blood-derived samples, leading to false negatives or inaccurate quantification. Digital PCR (dPCR), which partitions samples into thousands of independent microreactions, mitigates these issues by providing markedly enhanced analytical sensitivity and greater inhibitor tolerance. Comparative studies have shown that dPCR enables reliable quantification of bacterial DNA at very low concentrations where qPCR fails and offers superior precision and reproducibility [85]. Consequently, dPCR has emerged as a more robust tool for clinical investigations of low-level bacterial infections—such as those exploring the microbial contribution to thrombotic events—by delivering more reliable and sensitive detection than conventional molecular techniques.

Despite these advances, studies investigating the link between oral bacteria and stroke are few in number, have small sample sizes, and focus on highly specific populations with particular lifestyle or demographic characteristics. Consequently, these limitations restrict both the generalizability of the findings and the ability to establish causal relationships. Future research should include larger and more diverse cohorts, integrate functional analyses to better clarify potential mechanisms, and employ robust detection methods such as dPCR to improve sensitivity and accuracy.

## 3. Biological Mechanistic Hypothesis Linking Periodontal Bacteria and Ischemic Stroke

**Figure 1:**
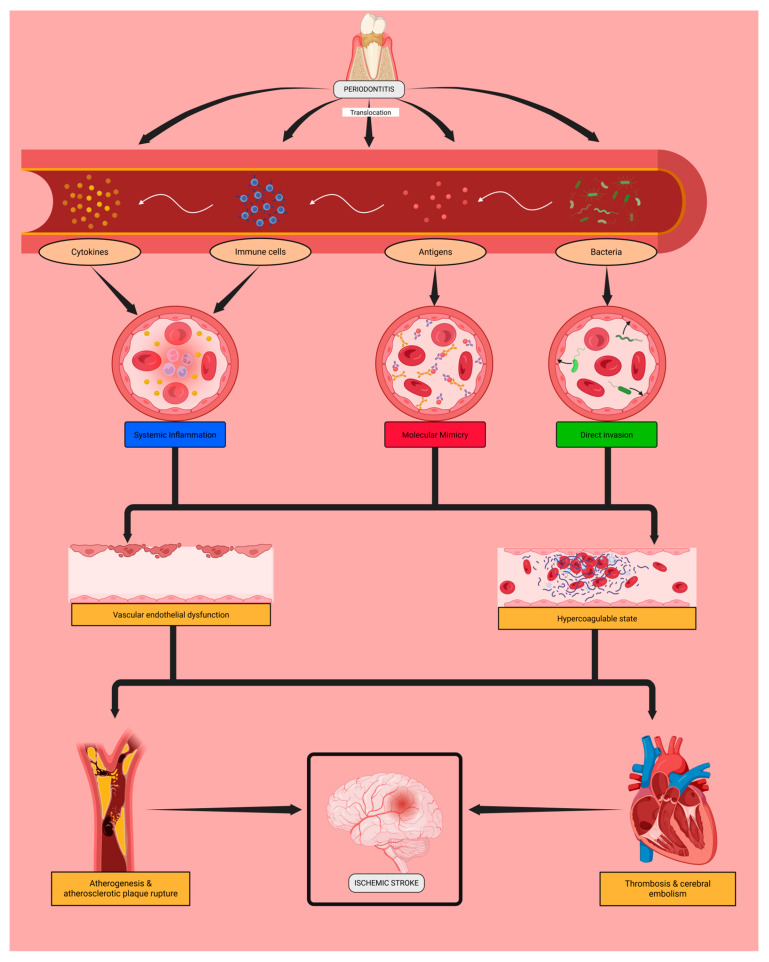
Potential mechanisms underlying the association between periodontal infection and cerebral ischemia. Periodontal pathogens may enter the bloodstream and trigger inflammatory, immune-mediated, or direct vascular effects that could promote endothelial dysfunction and prothrombotic changes, potentially contributing to atherothrombotic or cardioembolic stroke. Created in Biorender. Moldes, J. (2025). https://app.biorender.com/illustrations/68e954e867610443896cb7fe (accessed on 6 November 2025).

The mechanistic hypothesis linking periodontitis and ischemic stroke is based on the systemic effects of periodontal pathogens (Figure 1).

These oral bacteria may induce inflammation in the supporting dental tissues, leading to their destruction and the translocation of bacteria, bacterial antigens, and reactive inflammatory cells and cytokines into the bloodstream. This process could contribute to the progression of local periodontal disease and may be associated with systemic complications.

Periodontopathogens might directly invade the vascular endothelium, inducing its activation and dysfunction. Microbial antigens are recognized by immunoglobulins (such as IgA and IgG), which may interact with endothelial proteins and further enhance endothelial activation and injury. In parallel, immune cells and the cytokines they release in response to these microorganisms could sustain a chronic low-grade systemic inflammatory state that may contribute to endothelial dysfunction.

Endothelial damage throughout the cardiovascular system may favour subendothelial atheroma deposition and cardiac fibrosis, promoting unstable atherosclerosis and the development of atrial fibrillation. Ultimately, these intermediate conditions could contribute to plaque formation and rupture as well as cerebral thrombosis and embolism, the main etiological mechanisms of ischemic stroke—specifically of the atherothrombotic and cardioembolic subtypes.

Future studies should aim to validate these mechanistic links experimentally by integrating microbiological, immunological, and vascular assessments. Longitudinal and interventional designs, including microbiome profiling, inflammatory and endothelial biomarkers, and vascular imaging, will be essential to determine the causal contribution of periodontal pathogens to endothelial dysfunction and cerebrovascular events.

## 4. Conclusions

This review highlights the current evidence regarding the presence of oral bacteria and their products in stroke patients. Although oral bacteria DNA has been detected in thrombi, no data specifically demonstrate the presence of periodontal pathogens in thrombus samples. However, antibodies against periodontal pathogens have been detected both in serum and thrombotic samples of stroke patients, supporting a potential link between periodontitis and ischemic cerebrovascular disease.

Clinically, confirming this connection could have major implications for stroke prevention and patient management. Periodontal evaluation and treatment might emerge as a relevant strategy in order to reduce vascular inflammation and stroke recurrence. Early identification of patients with high-risk oral microbiota profiles or residual inflammatory risk could also support personalized preventive strategies in cerebrovascular patients.

## Figures and Tables

**Table 1:** Studies looking at the presence of oral bacteria on thrombi from ischemic stroke patients.

Study	Population	Demographic Characteristics	Type of Stroke (TOAST)	Retrieval Procedure	Microbial Methodology	Relevant Oral Bacteria	Main Findings	Ref.
Patrakka et al. 2019	75 patients with ischemic stroke (Tampere University Hospital, Finland)	- Mean age: 66.9 ± 12.4 years - Gender: 69.3% male - Hypertension: 53.3% - Dyslipidemia: 38.7% - Diabetes: 16% - AF: 66% - Smoking: 34.9%	- LAA: 62% - CE: 38%	Endovascular thrombectomy	Quantitative PCR	*Streptococcus* spp. (*S. mitis*) *P. gingivalis* *A. actinomycetem-comitans*	- Bacterial DNA was found in 84% of cerebral thrombi; *Streptococcus* spp. in 78.9% (mainly *S.mitis*). - No thrombi contained *P. gingivalis* or *A. actinomycetemcomitans*. - In arterial blood samples: 7 were positive for *Streptococcus* spp. (9.33%), 1 for both *P. gingivalis* and *A. actinomycetemcomitans* (1.33%).	[74]
Yadav et al. 2023	14 patients with ischemic stroke (Pacific Medical University, India)	- Mean age: 52 ± 13.6 years - Gender: 72% male - Hypertension: 50% - Diabetes: 21.4%	- LAA: 100%	Endovascular thrombectomy	16S rRNA sequencing and metagenomics analysis (spectrophotometry)	*Streptococcus* spp. *Prevotella* spp. *Lactobacillus* spp. *Veillonella* spp. *Dialister microaerophilus* *Sneathias* spp.	- 100% of thrombi had bacterial DNA. - Over 30 bacterial species identified. - Most common bacteria detected: *Staphylococcus aureus* (12%) *Streptococcus pneumoniae* (12%) *Lactobacillus* spp. (11%) *Bacillus cereus* (10%) *Bacillus subtilis* (5%) - Oral bacteria detected: *Streptococcus* spp. (pneumoniae, pyogenes) *Prevotella* spp. (*P. amnii*, *P. timonensis*, *P. buccalis*, *P. colorans*) *Lactobacillus* spp. (*L. crispatus*, *L. iners*, *L. jensenii*, *L. delbrueckii*, *L. gasseri*, *L. helveticus*) Veillonellaceae (*Veillonella* spp., *D. micraerophilus*) *Sneathia* spp. (*S. sanguinegens*, *S. amnii*) - Other opportunistic pathogens: *Staphylococcus* spp. (*S. aureus*, *S. epidermidis*, *S. saccharolyticus*) *Acinetobacter* spp. *Pseudomonas* spp. *Stenotrophomonas* spp. *Enterococcus faecium* - Higher levels of *Acinetobacter* spp. and *Enterobacteriaceae* were associated with early adverse events and poor prognosis.	[75]
Liao et al. 2022	104 patients with ischemic stroke (The First Affiliated Hospital, Jinan University, China)	- Median age: 65 years - Gender: 57.7% males - Hypertension: 68.2% - Diabetes: 33.7% - AF: 37.5% - Smoking: 39.4% - Alcohol: 15.4%	- LAA: 56.7% - CE: 43.3%	Endovascular thrombectomy (June 2019–June 2020)	16S rRNA sequencing and FISH	*Prevotella* spp. *Streptococcus* spp. *Lactobacillus* spp. *Veillonella* spp.	- 104 thrombus samples identifying 14 bacterial phyla, 182 genera, and 542 ASVs (Amplicon Sequence Variants) (>0.1% abundance). - Dominant phylum: Proteobacteria (73.3%), Bacteroidetes (12.9%), Firmicutes (10.0%) and Actinobacteria (2.0%). - Top genera included Acinetobacter (12.8%), Burkholderia (12.5%), Sphingomonas(10.9%), Pedobacter (7%), Serratia (5.5%) and Stenotrophomonas (5.3%). - Oral bacteria detected: *Streptococcus* spp. *Prevotella* spp. Veillonellaceae - Other opportunistic pathogens: *Staphylococcus* spp. *Acinetobacter* spp. *Stenotrophomonas* spp. - Thrombus microbiota closely resembled plasma microbiota; minor contribution from oral (2.3%) and fecal (2.1%) microbiota. - *Lactobacillus* spp. and *Chryseobacterium* spp. were more abundant in thrombi from LAA patients. Veillonellaceae was more frequent in thrombi from CE stroke. - During 90-day follow-up, 16.3% of patients died. Death group showed higher white blood cell counts (WBC), IL-1β, and IL-6 levels (significant differences) and higher abundance of *Acinetobacter* spp. and *Enterobacteriaceae* in thrombi. - *Acinetobacter* spp. abundance significantly associated with 90-day mortality. - No significant association between oral/fecal microbiota and mortality. - Findings suggest thrombus microbiota (especially *Acinetobacter* spp.) and systemic inflammation are linked to poor stroke outcomes.	[76]
Wang et al. 2024	81 patients with ischemic stroke (Jinling Hospital, China)	- Median age: 70 years - Gender: 64.2% male - Hypertension: 65.4% - Diabetes: 23.4% - AF: 60.5% - Smoking: 29.6% - Alcohol: 23.4%	- LAA: 24.7% - CE: 55.6%, - Others: 19.8%	Endovascular thrombectomy	- 16S rRNA sequencing - Transmission electron microscopy - Immunohistochemistry (CD14 marker)	*Streptococcus* spp. *Prevotella* spp. *Corynebacterium* spp.	- Bacterial DNA (16S rRNA) in 32.1% of thrombi. - Presence of oral bacterial DNA with statistical difference with blood control samples: *Streptococcus* spp. (1.53% vs. 0.29%, *p* = 0.001), *Prevotella* spp. (1.57 vs. 0.38%, *p* = 0.010), *Corynebacterium* spp. (1.61% vs. 1.26%, *p* = 0.026). - Transmission electron microscopy revealed partial and whole bacteria-like structures in 27.1% and 8.6% of thrombi, respectively. - CD14^+^ marker positive in 63% of thrombi. - Alcohol linked to higher bacterial DNA in thrombi (38.5% vs. 16.4%; OR 3.19; *p* = 0.033).	[77]
Patrakka et al. 2023—BMG	61 patients with ischemic stroke (Tampere University Hospital, Finland)	- Mean age: 66.8 ± 10.9 years - Gender: 70.5% male - Hypertension: 50.8% - Dyslipidemia: 39.3% - Diabetes: 18% - AF: 63.9% - Smoking: 36.1%	- LAA: 62% - CE: 38%	Endovascular thrombectomy	- Quantitative PCR - Histology: immunostained with antibody cocktail containing antibodies against streptococci (*S. sanguinis*, *S. mitis*, and *S.gordonii*)	- PCR: *Streptococcus* spp. (*S. mitis*) *P. gingivalis* *A. actinomycetemcomitans* - Histology: *S. viridans* (*S. mitis* and *S. sanguinis*) *S. gordonii*	- *Streptococcus* spp. DNA was detected in 78.7% of thrombi. - No DNA from *P. gingivalis* or *A. actinomycetemcomitans* was found in thrombi. - In control arterial blood samples: *Streptococcus* spp. in 9.33% *P. gingivalis* and *A. actinomycetemcomitans* DNA in 1.33% each. - 39 samples (84.8%) were histologically positive for viridans strep-tococci group bacteria.	[78]
Freiherr von Seckendorff et al. 2024	175 consecutive thrombi (Rothschild Foundation Hospital, Paris 2016–2018)	- Mean age: 71 years - Gender: 52% male - Hypertension: 57.7% - Dyslipidemia: 26.1% - Diabetes: 19.4% - Smoking: 15.4%	- CE: 53.1% - LAA: 13.1% - Stroke of other determined etiology: 5.1% - Stroke of undetermined etiology: 27.4%	Endovascular thrombectomy: stent-retriever and/or a contact aspiration technique	Inmunostaining with a commercial antibody that recognizes specifically gingipain/hemagglutinin of *P. gingivalis*	*P. gingivalis*	- *P. gingivalis* antigen was found in 33.6% of ischemic stroke thrombi. - More missing teeth in *P. gingivalis* (median 5 vs. 3). - Lower hypercholesterolemia rate in *P. gingivalis* (18.6% vs. 30.7%) - Less internal carotid artery occlusion in *P. gingivalis* group (23.7% vs. 41.4%). - Less intravenous thrombolysis use in *P. gingivalis* (35.6% vs. 56.0%). - No differences in prestroke disability, stroke severity, or etiology - *P. gingivalis* thrombi had lower complete reperfusion rate after treatment (39.0% vs. 57.8%). - Higher neutrophil elastase in *P. gingivalis* (median 180 vs. 129) - Trend toward worse 90-day functional outcome in *P. gingivalis* patients (35.7% vs. 49.5%), not statistically significant.	[79]

AF: atrial fibrillation. LAA: large artery atherosclerosis. CE: cardioembolic. FISH: Fluorescence in situ hybridization. Ref: reference.

## Data Availability

No new data were created or analyzed in this study. Data sharing is not applicable to this article.

## Abbreviations

The following abbreviations are used in this manuscript:

AF	Atrial Fibrillation
CAL	Clinical attachment loss
CE	Cardioembolic
CI	Confidence Interval
CRP	C-reactive protein
DALYs	Disability-adjusted life years
FISH	Fluorescence in situ hybridization
HSPs	Heat shock proteins
Ig	Immunoglobulin
IL	Interleukin
LAA	Large artery atherosclerosis
LPS	Lipopolysaccharide
OR	Odds Ratio
PCR	Polymerase chain reaction
TNF	Tumor necrosis factor
